# Sub-threshold or full-syndrome borderline personality disorder in adolescents with recurrent self-harm – distinctly or dimensionally different?

**DOI:** 10.1186/s40479-023-00234-z

**Published:** 2023-09-14

**Authors:** Anne Brager-Larsen, Pål Zeiner, Lars Mehlum

**Affiliations:** 1 Child and Adolescent Mental Health Research Unit, Division of Mental Health and Addiction, Department of Research and Innovation, Oslo University Hospital, Sognsvannsveien 12, Bygg 12, N-0372 Oslo, Norway; 2 National Centre for Suicide Research and Prevention, Institute of Clinical Medicine, University of Oslo, Oslo, Norway

**Keywords:** Borderline personality disorder (BPD), Sub-threshold, Self-harm, Non-suicidal self-injury (NSSI), Suicide attempt, Adolescents

## Abstract

**Background:**

Borderline personality disorder (BPD) is a severe mental disorder frequently seen in individuals with recurrent self-harm behaviour. To what extent there are distinguishing characteristics between self-harming adolescents who meet the criteria for a full diagnosis of BPD, a sub-threshold number of BPD criteria and those who don’t have BPD, with respect to clinical characteristics, is still uncertain and could have important clinical implications.

**Methods:**

Data from 103 adolescents with recurrent self-harm behaviour recruited from child and adolescent psychiatric outpatient clinics were collected through clinical interviews and self-reports. Bivariate analyses comparing participants with or without a diagnosis of BPD were performed. Group differences based on the number of BPD criteria fulfilled (few-if-any BPD: 0–2 criteria, sub-threshold BPD: 3–4 criteria, full-syndrome BPD: 5 or more criteria) were tested and regression analyses performed.

**Results:**

Adolescents with a diagnosis of BPD (28.2%) had significantly higher numbers of co-morbid DSM-5 disorders, suicide attempts and self-harm methods. They also reported significantly higher levels of suicidal ideation, depression, anxiety and impulsivity, compared with adolescents without BPD. Adolescents with sub-threshold BPD (20.4%) place themselves in the intermediate position between participants with full-syndrome BPD and participants with few-if-any BPD, in terms of these symptoms. Higher levels of emotional regulation difficulties and a lower level of global functioning were significantly associated with fulfilling a higher number of BPD criteria.

**Conclusion:**

Adolescents with recurrent self-harm who meet diagnostic criteria for a full-syndrome BPD or sub-threshold BPD seem to have difficulties within the same spectrum. They seem dimensionally, but not categorically, different with respect to the severity of their difficulties. These adolescents need interventions aimed at their dysfunctional self-harm behaviour, emotional regulation difficulties and BPD symptoms at an earlier, rather than at a later stage of symptom development.

## Introduction

Borderline personality disorder (BPD) is a severe mental health disorder frequently seen in individuals with recurrent suicide attempts and non-suicidal self-injury (NSSI); these are both behaviours subsumed under the broader term ‘self-harm’ [1]. BPD is mainly characterized by features such as unstable emotion regulation and interpersonal relationships, poor impulse control and an unstable self-image, in addition to the recurrent self-harm [2]. Among adolescents seen in mental health care settings, BPD has an estimated prevalence of 11% among outpatients [3], and up to 50% among inpatients [4, 5]. As with recurrent self-harm behaviour, BPD symptoms usually first appear in early adolescence, peak in late adolescence and early adulthood, and decline thereafter [6, 7].

Although recurrent self-harming behaviour is a frequently observed feature of BPD in adolescence [8], previous studies suggest that this is neither a necessary nor a sufficient feature for diagnosing BPD [9, 10]. However, in a recent review Reichl and Kaess [8] point out that NSSI is a relatively easily observable risk factor for early detection of BPD, which could imply that clinicians who detect recurrent self-harm behaviour in their adolescent patients should be alert to the possibility that these patients may have difficulties consistent with BPD. This will have important implications for choice of treatment. Despite the substantial clinical importance of gaining more knowledge of the association between self-harm behaviour and BPD, how these problems emerge in adolescence and how they could be detected and treated, published research in this field is currently relatively scarce. Of particular interest is gaining knowledge of possible similarities or differences between having a full-syndrome BPD and a sub-threshold BPD in adolescents with recurrent self-harm behaviour.

There is accumulating evidence that BPD often has it’s onset in the period between puberty and late teens and that many of the problems associated with a full-syndrome BPD emerge early in the course of the disorder, and that diagnosis and treatment are rarely offered at such an early stage [11]. This is disconcerting given that BPD can be reliably diagnosed and treated in adolescents in much the same way as in adults [12–14]. Studies show that adolescents with BPD have lower levels of global functioning, higher rates of comorbid mental disorders, (such as mood disorders), compared to adolescents with non-BPD mental disorders [15, 16]. Furthermore, studies of both adolescents and adults have shown that emotional dysregulation, broadly defined as the inability to flexibly respond to and manage emotions [17], is a common feature of both self-harming behaviour and BPD [18, 19]. It has also been suggested that facets of emotional dysregulation are observable during childhood and adolescence, before the emergence of self-harm behaviour and BPD symptoms [20].

In recent years, research has shown that early symptoms of BPD in adolescents are unlikely to disappear without intervention, suggesting that clinicians should systematically screen for these symptoms [11]. The phenomenological definition of BPD is today based on a dichotomous cut-off of five or more BPD criteria [2]. However, it has long been argued that BPD may be better understood as difficulties along a dimension of severity [21]. A compromise has been to adopt a 3-point dimensional convention (few-if-any: 0–2, sub-threshold: 3–4, full-syndrome BPD: 5 or more BPD criteria), which is equally valid as more fine-grained approaches, according to Zimmerman et al., [21]. So far only a few studies have looked at the differences in the number of BPD criteria met. These studies found that a sub-threshold number of BPD features are clinically meaningful indicators of psychopathology and poor psychosocial functioning [15, 22]. However, these studies have been based on adult samples or mixed samples of adolescents and adults [22, 23], reporting results which cannot be immediately translated into adolescent clinical populations. Kaess et al., [24], examined the number of BPD criteria in a group of risk-taking and/or self-harming adolescents, and found that subjects with sub-threshold BPD had impairments on comparable levels as adolescents with a full-syndrome BPD. However, this study was based on data from a specialized outpatient clinic only recruiting patients with any recent engagement in risk-taking behaviour (such as binge-drinking, substance abuse, excessive media/internet use, high risk sexual behaviour or delinquent behaviour) and/or self-harm behaviour, and results may not be easily generalizable to other adolescent clinical populations.

To the best of our knowledge, there is still a limited amount of studies in clinical adolescent populations with recurrent self-harm behaviour and BPD features, and especially studies that focus on differences between sub-threshold BPD and full-syndrome BPD difficulties. Although more research has recently emerged finding that BPD starts early and that it is possible to diagnose BPD in adolescence, these are mainly studies from adult samples, and studies that focus on differences between having a diagnosis of BPD or not. In adolescent outpatient clinics, most clinicians will at some point meet children and adolescents with recurrent self-harm behaviour and BPD features, and they need relevant and updated knowledge about the population they will encounter. Thus, there is a need of studies from clinical adolescent samples, with recurrent self-harm behaviour and with varying degrees of BPD difficulties, which could have important implications for treatment planning.

In the current study we adopted a 3-point dimensional convention (few-if-any BPD: 0–2 criteria, sub-threshold BPD: 3–4 criteria, full-syndrome BPD: 5 or more criteria), and utilized the regular DSM-5 algorithm [2] for the full diagnosis of BPD. Thus analysing BPD pathology both categorically and dimensionally in a clinical sample of adolescents with recurrent self-harming behaviour, we aimed to examine the extent to which there are distinguishing characteristics between adolescents who meet criteria for a full-syndrome BPD, a sub-threshold number of BPD criteria and those who are without BPD, with respect to self-harm behaviour and related clinical characteristics.

## Methods

### Participants and procedures

Participants were 103 adolescents (age 12—18) recruited from a child and adolescent psychiatric outpatient clinic at Oslo University Hospital, Norway. Inclusion criteria were recurrent self-harm behaviour (two or more episodes) with the last episode within the past 6 months. Exclusion criteria were intellectual disability or insufficient Norwegian language skills to participate in the study. Potential participants were screened for self-harm behaviour defined as** “**intentional poisoning or self-injury, regardless of intention to die” [25]. Non-suicidal self-injury (NSSI) was defined as “the deliberate, self-inflicted destruction of body tissue without suicidal intent, and for purposes not socially sanctioned” [26], while suicide attempt was defined as “a potentially self-injurious act committed with at least some wish to die, as a result of the act” [27]. The adolescents and their parents provided written informed consent and the study was approved by the Regional Committee for Medical Research Ethics, South-East Norway. Interviewers were experienced clinicians, having received training and supervision in the use of the study instruments.

### Measures

Information on frequency of non-suicidal self-injury episodes (NSSI), suicide attempts and self-harm methods in use, age of self-harm onset and duration of self-harm behaviour, was collected with the *Suicide Attempt Self-Injury Interview (SASII)*; a robust and comprehensive instrument with good psychometric properties [28, 29]. DSM-5 Axis I diagnoses were assessed with the *Schedule for Affective Disorders and Schizophrenia for School-Age Children-Present and Lifetime Version, 2016 (K-SADS-PL)* [30] whereas Borderline personality disorder was assessed with the *Childhood Interview for Borderline Personality Disorder (CI-BPD*), [31]; a semi-structured interview developed specifically for use with children and adolescents, with good psychometric properties [32]. The 9 criteria reflecting symptoms of BPD were rated with ‘0’ for absent symptoms, ‘1’ if the symptom were likely to be present and ‘2’ for symptoms that were definitely present. A BPD diagnosis requires a minimum of 5 criteria fulfilled with a score of ‘2’. An interrater reliability check of a random sample of participants (*n* = 13) was performed by two independent raters, resulting in a kappa value of 0.82 for the BPD diagnosis, which is generally considered a satisfactory level of reliability [33]. Testing the reliability of each of the BPD criteria, yielded a mean ICC (1,1) of 0.70 (range = 0.44–1.00), indicating moderate to good reliability [34].

The level of global psychosocial functioning was assessed by the *Children’s Global Assessment Scale* (CGAS) [35] with a score in the range of 1–100. Interviewer-rated level of depression was measured by the *MADRS* [36], whereas self-reported depressive symptoms were measured by the 13-item version of the Mood and Feelings Questionnaire (SMFQ) [37].

Borderline symptoms were assessed through the 23-item self-report *Borderline Symptom List (BSL-23*), [38], tapping difficulties and problems commonly experienced by people with BPD, e.g., “I hated myself”, “I didn’t trust other people”, “I wanted to punish myself” and “Criticism had a devastating effect on me”. Level of emotional dysregulation symptoms was measured with the 36-item self-report *Difficulties in Emotion Regulation Scale* (DERS), [39], whereas the level of impulsivity was measured with the 15-item self-report *Barratt Impulsiveness Scale* (BIS-15), [40]. Level of suicidal ideation was measured by the 15-item self-report *Suicidal Ideation Questionnaire* (SIQ-Jr) [41]. Level of anxiety symptoms was measured by the 6-item version of the self-report *Hopkins Symptom Checklist* (HSCL) [42].

### Data analysis

Means and standard deviations are given for normally distributed variables, while median and inter-quartile ranges are given for variables with non-normal distribution. As is commonly observed in clinical studies of self-harm, number of NSSI episodes and suicide attempts are not normally distributed and had several extreme outliers, hence these variables were winsorized at the 90 percentile to test whether this would significantly change the mean and variance [43], which it did not (data not shown).

First, bivariate analyses were performed based on whether the participants met the criteria for a diagnosis of BPD or not, and differences between the two groups were tested using chi-square for categorical variables and independent-sample t-tests for continuous variables. Effect sizes with Cohen's d for sample means and Phi for bivariate statistics were calculated. Second, based on the number of fulfilled BPD diagnostic criteria, participants were analysed in the following three subgroups: 1) participants with few-if-any BPD (fulfilling 0–2 BPD criteria), 2) participants with sub-threshold BPD (fulfilling 3–4 BPD criteria) and 3) participants with full-syndrome BPD (fulfilling 5–9 BPD criteria). One-way ANOVA with post-hoc test for multiple comparisons and Bonferroni correction was performed to compare the effect of the number of BPD criteria met on the selected variables. Finally, logistic and linear regression analyses were performed, and variables with *p*-value less than 0.05 were selected for inclusion in these analyses. The selection of variables was further supported by the correlation matrix and collinearity tests (data not shown) indicating no severe signs of multi-collinearity. All tests were two-sided, and the significance level was set to 0.05. Analyses were performed with SPSS 27.0.

## Results

A total of 103 adolescents with a mean age of 15.9 years (SD = 1.47), the majority (86.4%) identifying as female, participated in the study. There were no significant differences in age of self-harm onset or age between groups. Participants received a mean number of 3.2 (range = 0–8) DSM-5 diagnoses with mood disorders and anxiety disorders being the most prevalent (Table 1). Fifty adolescents (48.5%) reported lifetime suicide attempt(s), of which twenty-seven (26.2% of the total sample) had more than one attempt. The lifetime frequency of NSSI episodes ranged from 0 to 990 (median = 50; IQR = 126). Cutting was the most commonly reported self-harm method (93.2%), followed by self-battering (41.7%), and stabbing (16.3%). Mean number of self-harm methods was 2.7 (range = 1–10). For details on characteristics of clinical variables, psychopathology and self-harm, see Tables 1 and 2.

**Table 1:** Sociodemographic and diagnostic characteristics of adolescents with recurrent self-harm (*N* = 103)

	**n**	**%**
Gender (females)	89	86.4
Age, mean (years) (SD = 1.47) 15.9		
Born in Norway	87	84.5
One or both parents born in foreign countries	43	41.7
Living with	39	37.9
- both parents		
- alternating between parents	15	14.6
- single parent	34	33.0
**Current DSM-5 diagnosis**^a^		
Any Mood Disorder	87	84.5
- Major Depressive Episode	59	57.3
- Dysthymia	7	6.8
- Unspecified Depressive Disorder	23	22.3
- Bipolar Disorder	4	3.9
Any Psychotic Disorder	13	12.6
Any Anxiety Disorder	76	73.8
PTSD	15	14.6
Any Eating Disorder	18	17.5
Any Substance-Use Disorder	10	9.7
ADHD	24	23.3
Borderline Personality Disorder	29	28.2
Any other diagnoses^b^	27	26.2

^a^Not mutually exclusive categories

^b^Conduct disorder, Oppositional defiant disorder, Unspecified disruptive behaviour disorder, Chronic motor or Vocal tic disorder, Autism spectrum disorder, Adjustment disorder

*PTSD* Post-traumatic stress disorder, *ADHD* Attention-deficit/hyperactivity disorder

**Table 2:** Differences in clinical characteristics between adolescents with different number of BPD criteria fulfilled

	**Total** **sample**	**Without BPD**		**BPD**	**χ**^**2**^**/t-test**	**Phi/** **Cohen's *d***	***p***	***F***	**Multiple comparisons ANOVA Posthoc test, Sig**		
		Few-if-any BPD	Sub- thres hold BPD	full- syndrome BPD							
		0–2 Criteria	3–4 Criteria	5–9 Criteria					0–2 vs 3–4 criteria	0–2 vs 5–9 criteria	3–4 vs 5–9 criteria
	**N = 103**	***n* = 53 (51.4)**	***n* = 21 (20.4)**	**n = 29 (28.2)**							
Age, mean years (SD)	15.9 (1.5)	15.7 (1.5)	16.0 (1.3)	16.2 (1.4)	1.4	0.320	0.887	1.4			
**Current DSM-5 diagnoses**^a^											
Mood Disorder, any (%)	87 (84.5)	41 (77.4)	18 (85.7)	28 (96.6)	4.5	0.209	**0.037**	2.1			
Anxiety Disorder, any (%)	76 (73.8)	34 (64.2)	18 (85.7)	24 (82.8)	1.7	0.128	0.223	2.7			
Number of DSM-5 diagnoses, mean (SD)^b^	3.2 (1.6)	2.6 (1.5)	4.0 (1.0)	3.9 (1.5)	2.5	0.641	**0.014**	12.2	*	**	
**Self-harm characteristics**											
Age of self-harm onset, yrs, mean (SD)	13.1 (1.8)	13.3 (1.8)	13.0 (1.7)	12.9 (1.9)	-1.9	-0.174	0.061	0.6			
Duration of self-harm, yrs, mean (SD)	2.8 (2.1)	2.3 (2.1)	3.0 (1.7)	3.3 (1.9)	2.5	0.395	**0.012**	2.6			
Number of NSSI episodes, l i fetime, median (IQR)	50 (126)	26.0 (127)	50.0 (146)	91.0 (172)	1.7	0.279	0.095	1.0			
Number of self-harm methods, mean (SD)	2.7 (1.6)	2.4 (1.2)	2.4 (1.4)	3.3 (2.1)	2.3	0.609	**0.028**	3.9		*	
Number of suicide attempts, l i fetime, median (IQR)	0.0 (2.0)	0.0 (1.0)	1.0 (1.0)	2.0 (5.0)	2.3	0.703	**0.025**	5.1		*	
Suicide attempt, any *n* (%)	50 (48.5)	21 (39.6)	11 (52.4)	18 (62.1)	2.3	0.169	0.086	2.3			
**Level of psychopathological distress**											
Borderline symptoms (BSL-23), mean (SD) (*n* = 101)	35.1 (23.1)	28.7 (21.5)	32.7 (20.3)	49.7 (22.2)	3.1	0.930	**0.003**	8.8		**	*
Emotional regulation difficulties (DERS), mean (SD), (*n* = 98)	108.1 (26.9)	101.2 (26.0)	101.7 (24.4)	127.6 (21.1)	4.0	1.077	** < 0.001**	10.7		**	*
Impulsivity (Bi S-Brief), mean (SD) (*n* = 99)	18.0 (5.9)	15.9 (5.3)	19.7 (5.5)	21.0 (5.7)	2.7	0.722	**0.008**	8.9	*	**	
Suicide ideation (SIQ-jr), mean (SD) (*n* = 99)	35.3 (22.6)	32.8 (21.4)	27.3 (18.0)	46.2 (24.6)	2.2	0.693	**0.027**	5.2		*	*
Depression (MADRS), mean (SD) (*n* = 98)	20.1 (8.4)	18.8 (9.3)	20.7 (7.9)	22.1 (6.7)	0.6	0.331	0.506	1.4			
Anxiety symptoms, (SCL6), mean (SD) (*n* = 102)	8.6 (4.6)	7.6 (4.2)	7.7 (5.2)	11.2 (4.2)	2.6	0.807	**0.012**	6.6		*	*
Mood and feelings questionnaire (MFQ), mean (SD)	16.0 (6.7)	14.2 (7.4)	15.5 (6.2)	19.9 (3.7)	3.4	0.845	** < 0.001**	7.7		**	
Global functioning (C-GAS), mean (SD)	53.3 (9.2)	57.2 (9.2)	52.0 (7.6)	47.1 (6.3)	-3.9	-1.025	** < 0.000**	14.5	*	**	

*SD* Standard deviation, *IQR* Interquartile Range

^a^Not mutually exclusive categories

^b^"Number of DSM-5 diagnoses" is summarized without the diagnosis of BPD. NSSI, nonsuicidal self-injury. CGAS, Childrens global assessment scale. Analyzed with Pearson's Chi-square test with Fisher's Exact test, sig. (2-sided), and Independent sample t-test. Effect size: Cohen's d for sample means, Phi for bivariate statistics. ANOVA between groups F-test. ANOVA post hoc test, multiple comarison based on means, with Bonferroni correction

^**^p < 0.001; *p < 0.05

Significant differences were mostly found between adolescents with a diagnosis of BPD and adolescents without BPD, as shown in Table 2. Participants with a BPD diagnosis received a significantly higher number of comorbid DSM-5 diagnoses compared with adolescents without BPD; notably more adolescents with BPD had a mood disorder than adolescents without BPD. Also, adolescents with BPD reported to have made a significantly higher number of suicide attempts, had significantly longer duration of self-harm behaviour and used a significantly higher number of self-harm methods, compared to adolescents without BPD, (Table 2).

Multiple comparisons were made between three subgroups of participants based on their number of fulfilled BPD, and differences were mostly found between adolescents with a full-syndrome BPD and adolescents with few-if-any BPD (Table 2). When compared with adolescents with few-if-any BPD, adolescents with a full-syndrome BPD had significantly higher number of comorbid DSM-5 diagnoses, higher number of suicide attempts and used a significantly higher number of self-harm methods. When compared with adolescents with sub-threshold BPD, adolescents with a full-syndrome BPD had significantly higher levels of emotion regulation difficulties, suicidal ideation, anxiety and borderline symptoms measured by BSL-23. However, no significant differences between these two sub-groups were found in self-harm characteristics or levels of global functioning, depressive symptoms (both clinician-rated and self-reported) or impulsivity.

Adolescents with sub-threshold BPD when compared with adolescents with few-if-any BPD, received a significantly higher number of DSM-5 diagnoses, and had a lower level of global functioning and higher level of impulsivity. There were no significant differences between these two sub-groups in terms of self-harm characteristics, emotional regulation difficulties, depressive symptoms (both clinician-rated and self-reported), anxiety symptoms, suicide ideation or borderline symptoms measured by BSL-23.

Finally, in the logistic regression analysis (Table 3) a higher level of emotion regulation difficulties and lower level of global functioning significantly increased the likelihood of receiving a diagnosis of BPD. Since BPD may be understood as difficulties along a dimension, we tested whether these variables were also associated with the total sum of BPD criteria met. A linear regression (Table 4) analysis confirmed the main findings from the logistic regression analysis, that an increasing level of emotional regulation difficulties and decreasing level of global functioning were significantly associated with an increase in the number of fulfilled BPD criteria.

**Table 3:** Multiple logistic regression for Borderline Personality Disorder (BPD) and selected variables

	*B*	Std.Error	Sig	*Exp(B)*
(Constant)	-1.33	3.54	0.71	0.26
Duration of self-harm	0.20	0.18	0.27	1.22
Number of DSM-5 diagnoses	0.32	0.23	0.15	1.38
Suicide attempt (lifetime)	0.13	0.11	0.26	1.13
Level of emotional regulation difficulties (DERS)	0.05	0.02	0.00	1.05
Level of impulsivity (BIS)	0.08	0.06	0.21	1.08
Level of depression (MADRS)	-0.12	0.06	0.03	0.88
Level of global functioning (CGAS)	-0.12	0.05	0.02	0.89

**Table 4:** Multiple linear regression for the sum of Borderline Personality Disorder (BPD) criteria and selected variables

	*B*	Std.Error	Sig	t
(Constant)	1.84	3.74	0.62	0.49
Duration of self-harm	0.41	0.18	0.03	2.26
Number of DSM-5 diagnoses	0.65	0.24	0.01	2.71
Suicide attempt (lifetime)	0.01	0.09	0.92	0.10
Level of emotional regulation difficulties (DERS)	0.05	0.01	0.00	3.36
Level of impulsivity (BIS)	0.17	0.06	0.01	2.83
Level of depression (MADRS)	-0.07	0.05	0.15	-1.45
Level of global functioning (CGAS)	-0.11	0.05	0.02	-2.37

Dependent variable based on number of BPD criteria; "0": absent. "1": likely present, "2": present

## Discussion

In this study of adolescents with recurrent self-harm behaviour, we aimed to examine the extent to which there are differences between adolescents with or without a BPD diagnosis, and between adolescents who fulfilled different numbers of BPD criteria (a full-syndrome BPD, a sub-threshold BPD and those with few-if-any BPD criteria), with respect to self-harm behaviour and related clinical characteristics. The three main findings of this study were that, a) the largest number of significant differences were found between adolescents with a diagnosis of BPD and adolescents without a BPD diagnosis, b) when comparing subgroups of adolescents with different number of fulfilled BPD-criteria, adolescents with a sub-threshold BPD (3–4 criteria) appeared as an intermediate group between adolescents with a full-syndrome BPD (5 or more) and adolescents with few-if-any BPD criteria (2 or less), and c) higher levels of emotion regulation difficulties and a lower level of global functioning were significantly and multivariately associated with a higher number of BPD criteria fulfilled.

Differences between adolescents with or without a diagnosis of BPD.

Adolescents with a diagnosis of BPD displayed significantly more signs of mental health problems compared with adolescents without BPD, such as a significantly higher number of comorbid DSM-5 disorders and a significantly lower level of global functioning. This corresponds well with earlier clinical studies suggesting that adolescents with BPD have more psychiatric comorbidity and are more severely impaired [16], compared with adolescents without BPD.

Also, in the current study, adolescents with a diagnosis of BPD reported a higher number of previous suicide attempts and a higher level of suicide ideation. Furthermore, they had used a higher number of self-harm methods, and had a significantly longer duration of self-harm behaviour than adolescents without BPD. These findings suggest that, even within a clinical sample of adolescents where all participants had recurrent self-harm behaviour, participants meeting criteria for a BPD diagnosis have significantly more severe suicidal and self-harming behaviour.

Differences between adolescents with full-syndrome BPD, sub-threshold BPD and few-if-any BPD criteria.

Adolescents with sub-threshold BPD reported significantly lower levels of emotion regulation difficulties, suicide ideation and anxiety symptoms compared with adolescents with full-syndrome BPD, but they had significantly more comorbid DSM-5 diagnoses and higher levels of suicide ideation than adolescents with few-if-any BPD criteria. Thus, adolescents with sub-threshold BPD place themselves in the intermediate position between participants with full-syndrome BPD and participants with few-if-any BPD criteria, in terms of these symptoms suggesting. This suggests that adolescents with sub-threshold BPD and full-syndrome BPD do not belong to categorically different groups, but rather dimensionally different sub-groups. The same can be said about the relationship between the sub-threshold group and the few-if any group, where only levels of impulsivity and global functioning were significantly different. However, it is important to keep in mind that all participants in this study sample had reported history of recurrent self-harm behaviour, signifying a high level of psychological distress. We don't know whether a group of adolescents without recurrent self-harm, but with few-if-any BPD criteria, would have been equally impaired.

Interestingly, we found no significant differences between adolescents with sub-threshold BPD and full-syndrome BPD in terms of number of NSSI episodes, number of suicide attempts or number of self-harm methods. We don't know yet exactly what roles self-harming behaviour may play in the pathogenesis of BPD. Hypothetically, an addictive pattern of self-harm as a strategy to regulate emotions may reinforce latent vulnerabilities among more sensitive and emotionally reactive adolescents and pave the way for development of other BPD related pathology. In this case, early detection of recurrent self-harm behaviour and a reduction of the duration of untreated self-harm could increase our ability to prevent, or even perhaps reverse, a process of developing a full BPD diagnosis through offering adequate treatment at an earlier stage. Our study cannot address this question empirically, and we are not aware of any research that has investigated this hypothesis. At the current state of knowledge, we would regard it important to target the group of adolescents with sub-threshold BPD that is very similar to full-syndrome BPD in many areas, for further study. However, we do not know how much of the sub-threshold symptoms will naturally persist or progress to full-syndrome BPD and how much will fade and disappear. Nor do we currently know how decisive it will be for the prognosis if these adolescents receive BPD-specific treatment, and only follow-up studies of this patient group will be able to answer this.

There is still a limited amount of studies in clinical adolescent self-harm populations that focus on differences between sub-threshold BPD and full-syndrome BPD difficulties. Thus, our study adds to the current knowledge concerning distinguishing characteristics between adolescents with recurrent self-harm behaviour and different number of BPD criteria fulfilled, and we believe this is important knowledge for clinicians regarding treatment planning for these patients.

In our multivariate analyses, we found that emotional dysregulation and global functioning were strongly predictive of BPD in this material. These findings are in accordance with previous studies [18, 19] and the way BPD is defined by its criteria, it is not surprising that we find this strong and consistent association also in this sample of self-harming adolescents. However, it suggests that early treatment should focus on strengthening emotional regulation skills and replace self-harm behaviour with more adaptive behaviours. In recent years, several disorder-specific psychotherapy treatments have been developed for adolescent with self-harm behaviour and BPD-features. Particularly dialectical behaviour therapy for adolescents (DBT-A) [44] and mentalization based therapy (MBT-A) [45] have shown positive effects of reducing self-harming behaviour among individuals with BPD features. In addition, more symptom-specific therapies targeting emotional regulation difficulties have been developed, [6]. Thus, there are good reasons for clinicians to early assess self-harm behaviour and BPD symptoms in adolescents, and offer BPD specific treatment.

## Strengths and limitations

Strengths of this study are that it is based on a clinical sample of adolescents with recurrent self-harm behaviour and that structured diagnostic interviews were used to assess all DSM-5 diagnostic criteria which enables reliable comparisons between studies. Interview-based assessments of BPD were checked for interrater reliability with satisfactory results. Among the limitations, are the study's cross-sectional design precluding longitudinal or causal conclusions. Also, the limited number of males (13.6%) included in the sample preventing us from generalizing these findings to males in clinical populations. Our findings, however, may be generalized to other similar clinical adolescent populations with recurrent self-harm behaviour. Future studies with more diverse samples and with a longitudinal design would improve confidence in the results, as well as generalizability to a broader clinical population.

## Conclusions

Consistent findings are that adolescents with recurrent self-harm behaviour with a diagnosis of BPD show a greater total burden of symptoms compared with the adolescents without BPD. Adolescents with sub-threshold BPD compared with full-syndrome BPD show difficulties much in the same areas, and seem dimensionally, but not categorically different with respect to the severity of their difficulties. Consequently, clinicians need to be aware that adolescents with recurrent self-harm behaviour may also have difficulties consistent within the BPD spectrum, and that these adolescents need interventions aimed at the recurrent self-harm behaviour and BPD specific difficulties at an earlier, rather than a later stage of symptom development.

## Data Availability

The datasets analyzed during the current study are available from the corresponding author on reasonable request. We have only used standard questionnaires and interviews that have been previously published elsewhere, and in according to guidelines.

## References

[1] Hawton K, Saunders KE, O'Connor RC (2012). Self-harm and suicide in adolescents. Lancet.

[2] American Psychiatric Association, Diagnostic and statistical manual of mental disorders : DSM-5. 5th ed. ed. DSM-5. 2013, Washington, D.C: American Psychiatric Association.

[3] Chanen AM (2008). Screening for borderline personality disorder in outpatient youth. J Pers Disord.

[4] Kaess M, Brunner R, Chanen A (2014). Borderline personality disorder in adolescence. Pediatrics.

[5] Grilo CM (1996). Gender differences in personality disorders in psychiatrically hospitalized adolescents. Am J Psychiatry.

[6] Bohus M (2021). Borderline personality disorder. Lancet.

[7] Plener PL (2015). The longitudinal course of non-suicidal self-injury and deliberate self-harm: a systematic review of the literature. Borderline Personal Disord Emot Dysregul.

[8] Reichl C, Kaess M (2021). Self-harm in the context of borderline personality disorder. Curr Opin Psychol.

[9] Glenn CR, Klonsky ED (2013). Nonsuicidal self-injury disorder: an empirical investigation in adolescent psychiatric patients. J Clin Child Adolesc Psychol.

[10] Neupane SP, Mehlum L. Adolescents With Non-Suicidal Self-Harm-Who Among Them Has Attempted Suicide? Arch Suicide Res. 2023;27(3):866–79. 10.1080/13811118.2022.2072254. 10.1080/13811118.2022.2072254 35579411

[11] Chanen AM (2015). Borderline Personality Disorder in Young People: Are We There Yet?. J Clin Psychol.

[12] Mehlum L (2014). Dialectical behavior therapy for adolescents with repeated suicidal and self-harming behavior: a randomized trial. J Am Acad Child Adolesc Psychiatry.

[13] Sharp C (2021). The Course of Borderline Psychopathology in Adolescents with Complex Mental Health Problems: An 18 Month Longitudinal Follow-up Study. Res Child Adolesc Psychopathol.

[14] Miller AL, Muehlenkamp JJ, Jacobson CM (2008). Fact or fiction: diagnosing borderline personality disorder in adolescents. Clin Psychol Rev.

[15] Thompson KN, Jackson H, Cavelti M, Betts J, McCutcheon L, Jovev M, Chanen AM. Number of Borderline Personality Disorder Criteria and Depression Predict Poor Functioning and Quality of Life in Outpatient Youth. J Pers Disord. 2020;34(6):785–98. 10.1521/pedi_2019_33_411. 10.1521/pedi_2019_33_411 30689518

[16] Chanen AM, McCutcheon L (2013). Prevention and early intervention for borderline personality disorder: current status and recent evidence. Br J Psychiatry Suppl.

[17] Carpenter RW, Trull TJ (2013). Components of emotion dysregulation in borderline personality disorder: a review. Curr Psychiatry Rep.

[18] Linehan MM (1993). Cognitive-behavioral treatment of borderline personality disorder. Diagnosis and treatment of mental disorders.

[19] Ford JD, Gomez JM (2015). The relationship of psychological trauma and dissociative and posttraumatic stress disorders to nonsuicidal self-injury and suicidality: a review. J Trauma Dissociation.

[20] Crowell SE, Beauchaine TP, Linehan MM (2009). A biosocial developmental model of borderline personality: Elaborating and extending Linehan's theory. Psychol Bull.

[21] Zimmerman M (2011). Does DSM-IV already capture the dimensional nature of personality disorders?. J Clin Psychiatry.

[22] Zimmerman M (2012). Does the Presence of One Feature of Borderline Personality Disorder Have Clinical Significance? Implications for Dimensional Ratings of Personality Disorders. J Clin Psychiatry.

[23] Thompson KN (2019). The Clinical Significance of Subthreshold Borderline Personality Disorder Features in Outpatient Youth. J Pers Disord.

[24] Kaess M (2017). Health related quality of life and psychopathological distress in risk taking and self-harming adolescents with full-syndrome, subthreshold and without borderline personality disorder: rethinking the clinical cut-off?. Borderline Personal Disord Emot Dysregul.

[25] Hawton K (2007). Psychiatric assessment and management of deliberate self-poisoning patients. Medicine (Abingdon 1995, UK ed).

[26] Klonsky ED (2007). The functions of deliberate self-injury: a review of the evidence. Clin Psychol Rev.

[27] Posner K (2007). Factors in the assessment of suicidality in youth. CNS Spectr.

[28] Borschmann R (2012). Measuring self-harm in adults: a systematic review. Eur Psychiatry.

[29] Linehan MM (2006). Suicide Attempt Self-Injury Interview (SASII): development, reliability, and validity of a scale to assess suicide attempts and intentional self-injury. Psychol Assess.

[30] Kaufman J, Birmaher B, Axelson D (2016). Axelson, Schedule for Affective Disorders and Schizophrenia for School-Aged Children: Present and Lifetime Version (K-SADS-PL) DSM-5 November 2016 working draft.

[31] Zanarini MC (2003). Zanarini rating scale for borderline personality disorder (ZAN-BPD): A continuous measure of DSM-IV borderline psychopathology. J Pers Disord.

[32] Sharp C (2012). Borderline personality disorder in adolescents: evidence in support of the Childhood Interview for DSM-IV Borderline Personality Disorder in a sample of adolescent inpatients. Compr Psychiatry.

[33] Landis JR, Koch GG (1977). The measurement of observer agreement for categorical data. Biometrics.

[34] Koo TK, Li MY (2016). A Guideline of Selecting and Reporting Intraclass Correlation Coefficients for Reliability Research. J Chiropr Med.

[35] Shaffer D (1983). A Children's Global Assessment Scale (CGAS). Arch Gen Psychiatry.

[36] Montgomery SA, Asberg M (1979). A new depression scale designed to be sensitive to change. Br J Psychiatry.

[37] Angold A (1995). Development of a short questionnaire for use in epidemiological studies of depression in children and adolescents. Int J Methods Psychiatr Res.

[38] Bohus M (2007). Psychometric properties of the Borderline Symptom List (BSL). Psychopathology.

[39] Gratz KL, Roemer L (2004). Multidimensional Assessment of Emotion Regulation and Dysregulation: Development, Factor Structure, and Initial Validation of the Difficulties in Emotion Regulation Scale. J Psychopathol Behav Assess.

[40] Meule A (2020). Confirmatory factor analysis of the Barratt Impulsiveness Scale-short form (BIS-15) in patients with mental disorders. Psychiatry Res.

[41] Reynolds WM, Mazza JJ (1999). Assessment of suicidal ideation in innercity children and young adolescents: reliability and validity of the suicidal ideation questionnaire–JR. School Psych Rev.

[42] Derogatis LR (1974). The Hopkins Symptom Checklist (HSCL). A measure of primary symptom dimensions. Mod Probl Pharmacopsychiatry.

[43] Field, A., Discovering statistics using IBM SPSS Statistics. 5th edition. ed. 2018, Los Angeles: SAGE.

[44] Miller AL, Rathus JH, Linehan MM (2007). Dialectical behavior therapy with suicidal adolescents.

[45] Fonagy P (2015). ESCAP Expert Article: borderline personality disorder in adolescence: an expert research review with implications for clinical practice. Eur Child Adolesc Psychiatry.

